# Giant Bilateral Hydronephrosis in A Newborn—A Case Report

**DOI:** 10.3390/children9121890

**Published:** 2022-12-02

**Authors:** Martina Frech-Dörfler, Sabrina Durand, Friederike Prüfer, Stefan Holland-Cunz, Christoph Rudin

**Affiliations:** 1 Department of Pediatric Surgery, University Children’s Hospital, 4031 Basel, Switzerland; 2 Department of Neonatology, University Children’s Hospital, 4031 Basel, Switzerland; 3 Department of Radiology, University Children’s Hospital, 4031 Basel, Switzerland; 4 Department of Nephrology, University Children’s Hospital, 4031 Basel, Switzerland

**Keywords:** giant hydronephrosis, newborn, ureteropelvic junction obstruction, CAKUT (congenital anomalies of the kidney and urinary tract), prenatal

## Abstract

Background: Prenatal hydronephrosis is common and may vary in size. Although mostly unproblematic, it may be a sign of urinary tract obstruction of differing severity. Case Diagnosis/Treatment: We present a boy with prenatally detected bilateral giant hydronephrosis. A prenatal ultrasound showed the whole abdominal cavity of the fetus filled with urine. Kidney parenchyma could not be seen. The boy was born at 34 + 1 weeks’ gestation. After delivery, he showed a severely distended abdomen. Insertion of a nasogastric tube was not possible, and he had to be intubated due to respiratory distress. A bilateral percutaneous nephrostomy was performed immediately. After a few hours, he could be stabilized and extubated. An ultrasound on the following day showed two kidney units with normal kidney parenchyma of normal size. The initially slightly elevated serum creatinine level normalized within one week. An antegrade pyelography via the nephrostomy tubes showed bilateral ureteropelvic junction obstruction. Conclusion: Severe bilateral hydronephrosis may be associated with good outcome and well-preserved kidney function. Prenatal counseling should be done carefully, with discussion of different treatment possibilities and without definitive prediction of outcome.

## 1. Introduction

Prenatal hydronephrosis is common and may vary in size. It occurs in 1–5% of all pregnancies and accounts for 50–87% of urinary tract anomalies [1,2]. There are multiple etiologies of hydronephrosis, including transient hydronephrosis, and obstruction at the level of the ureteropelvic or the vesicoureteral junction, posterior urethral valves or vesicoureteral reflux. In about 50% of cases, hydronephrosis is unproblematic and shows spontaneous resolution after birth. In around 25%, hydronephrosis is caused by urinary tract obstruction and has to be treated to prevent kidney damage on the affected side. Ureteropelvic junction obstruction (UPJO) reflects the most common reason for hydronephrosis requiring intervention [2].

Giant hydronephrosis in adults is defined as a hydronephrosis that either crosses the midline, involves the length of 5 vertebra, or contains more than 1 L of urine [3]. This correlates with 1.6% of bodyweight [4]. In children, hydronephrosis ranging from 4% of bodyweight at birth to 2% at puberty may be defined as giant hydronephrosis [5,6]. Giant hydronephrosis is rare and is found most frequently due to UPJO [7]. In general, immediate intervention after birth is needed to decompress the kidneys, as well as to improve ventilation. We would like to present a rare case of bilateral giant hydronephrosis with a poor prenatal prognosis, but an extraordinary outcome.

## 2. Case Presentation

**Figure 1:**
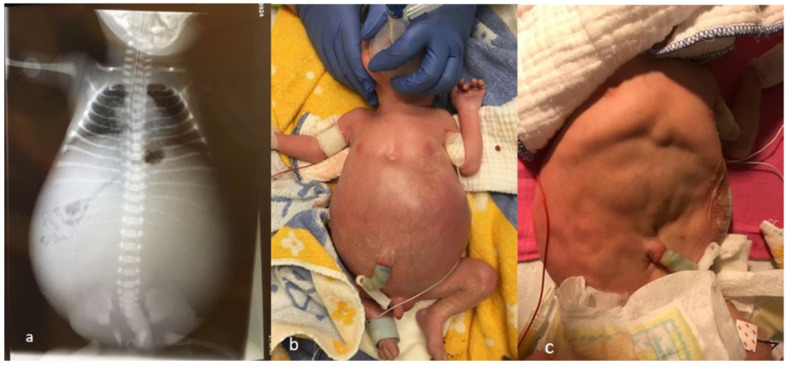
(**a**) Abdominal X-ray with compression of the intestine and the lung; (**b**) Distended abdomen before insertion of nephrostomy tube; (**c**) Distended abdomen after nephrostomy tube placement and evacuation of 1 L urine.

We hereby present a boy with prenatally identified bilateral giant hydronephrosis. Kidney parenchyma was not visible on the prenatal ultrasound. During pregnancy, hydronephrosis continued to increase. The amniotic fluid index (AFI) was normal until the 34th week of gestation and decreased shortly before birth. Prenatal counselling had been done at 19, 23 and 32 weeks gestation, respectively. Chromosomal testing didn’t show any abnormalities. Due to onset of labor, the boy was born at 34^+1^ weeks’ gestation. Postnatal respiratory support consisted of positive end-expiratory pressure (PEEP) ventilation and oxygen application. Apgar scores were 1, 7 and 7 at 1, 5 and 10 min, respectively. The birthweight was 3170 g, the length 47 cm and the head circumference 31 cm. Due to a severely distended abdomen (Figure 1b) ventilation was compromised, and he showed progressive respiratory failure that prompted immediate intubation. Positioning of a nasogastric tube was not possible. An abdominal X-ray showed displacement and compression of the intestine (Figure 1a).

Bilateral percutaneous nephrostomy tubes were inserted immediately at the neonatology ward. A total of around 1 L of urine (600 mL on the left, 400 mL on the right side) could be extracted, which accounted for 43% of his body weight. His weight hence decreased to 2300 g.

**Figure 2:**
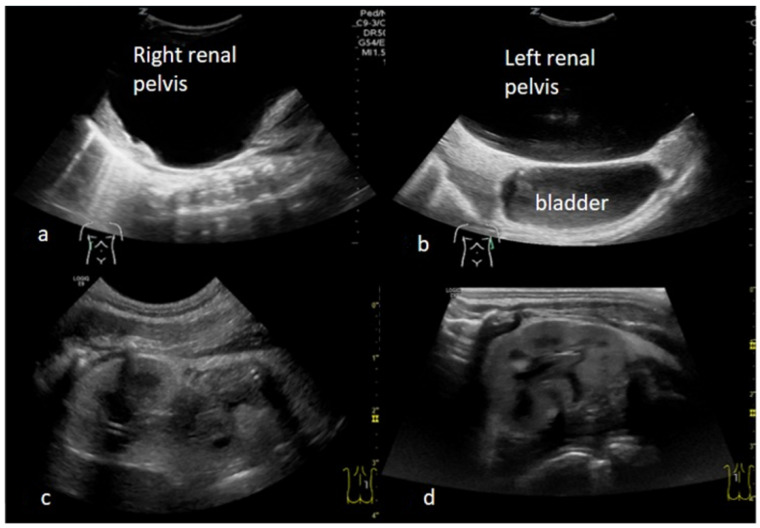
(**a**) Right renal pelvis before insertion of nephrostomy tube; (**b**) Left renal pelvis above the bladder before insertion of nephrostomy tube; (**c**) Right kidney after insertion of nephrostomy tube; (**d**) Left kidney after insertion of nephrostomy tube.

Within 12 h, the boy stabilized and could be extubated. Ultrasound showed two kidneys with normal parenchyma and of normal size (Figure 2a–d). The serum creatinine level was initially slightly elevated (max 113 mcmol/L), but normalized within the first week of life.

There was no evidence for additional malformations. Echocardiographic, abdominal and cerebral ultrasound studies were within normal limits during the first days of life. Due to his prematurity, nasogastric tube feedings were necessary during the first days of life. At the age of 14 days, he could be discharged on full oral feeds from hospital.

A voiding cystourethrogram performed 6 days after birth showed no vesicoureteral reflux and no signs of posterior urethral valves. An antegrade pyelography via the inserted nephrostomy tubes showed nearly complete obstruction at the ureteropelvic junction. At 4 weeks of age, a MAG3 renal scan confirmed the diagnosis of bilateral ureteropelvic junction obstruction. Split function was 11% on the right, and 89% on the left side.

At the age of 6 weeks, a bilateral Anderson–Hynes pyeloplasty was performed, and bilateral percutaneous stents were left in place. Ten days postoperatively, the percutaneous stents were closed. Ultrasound showed no increase in pelvis dilation on either side, and the stents were removed. Fourteen days later, there was increasing bilateral hydronephrosis. Nephrostomy tubes had to be reinserted on both sides, as insertion of double J-stents was not possible.

The further course showed an additional vesicoureteral junction obstruction on the left side, which had to be treated by ureteral re-implantation.

**Figure 3:**
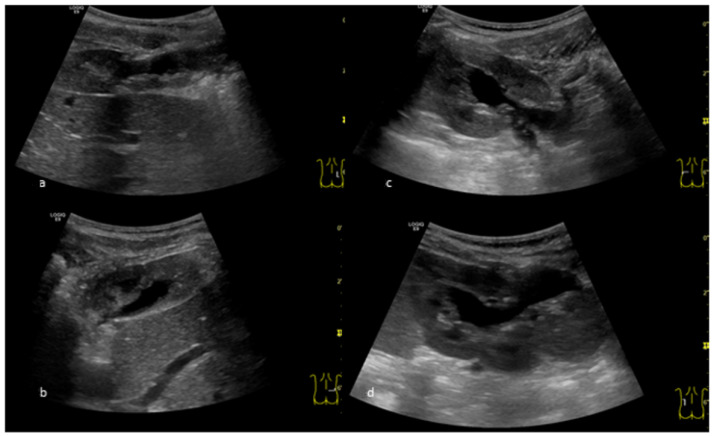
Ultrasound nearly 4 years postoperatively: (**a**) Right kidney: longitudinal view; (**b**) Right kidney: cross-sectional view; (**c**) Left kidney: longitudinal view; (**d**) Left kidney: cross-sectional view.

The nephrostomy tubes could be removed at the age of 7 months on the right, and at the age of 13 months the left side. Repeated ultrasounds continued to show dilation on both sides, but they remained stable and decreased slowly over the first years of life (Figure 3a–d). Another MAG 3 renogram at 19 months of life showed an improved split function of 30% on the right side. No urinary tract infections occurred up to the age of 3 years. Antibiotic prophylaxis had been stopped after the removal of the left nephrostomy tube (at 13 months of age). Serum creatinine has always been normal. Due to a high myopia—which had been detected at nearly two years of age—genetic testing was repeated, showing again no abnormalities. The boy shows age-appropriate psycho-motoric development.

## 3. Discussion

Congenital giant hydronephrosis is rare in newborns and even rarer if occurring bilaterally. There is not much literature about giant hydronephrosis in newborns, a vast majority being case reports of adults and older children. Shimada et al. describe a series of nine newborns with congenital giant hydronephrosis within a cohort of 562 patients with hydronephrosis over a time period of 13 years [8]. A study from Kaura reports 35 patients, of whom 16 were children, with giant hydronephrosis [3]. Another case report describes gastrointestinal obstruction caused by unilateral giant hydronephrosis in a newborn [9]. However, to the best of our knowledge, there are no reports of bilateral giant hydronephrosis in a newborn similar to what we have observed in our patient.

In the majority of cases, ureteropelvic junction obstruction has good long-term prognoses concerning kidney function [10,11]. Baek et al. reported a series of 30 children with giant hydronephrosis, who were treated with pyeloplasty due to UPJO. Only one of these patients had kidney failure, with a final residual kidney function of less than 10%. They concluded that primary nephrectomy is not justified [11]. Likewise, in our patient, severe hydronephrosis was not associated with a poor outcome. The initially bad split function of 11% on the right side improved up to 30% over time. Therefore, we also suggest that early nephrectomy should be avoided.

Most urinary malformations can be diagnosed by prenatal ultrasound including UPJO [12]. Typical findings for this condition are unilateral or bilateral dilation of the pelvis of different severity, classified according to different available grading systems. The Hydronephrosis grading system of the Society for Fetal Urology (SFU) and the Urinary Tract Dilation (UTD) classification system are most commonly used [12,13,14]. Another measurement parameter is the anterior–posterior diameter (APD). An APD of >15 mm is described as the cut-off for prediction of possible postnatal surgery, but is not a predictive marker for kidney function [13,15].

In patients with UPJO, the ureter usually is not dilated, and bladder size as well as bladder wall thickness are normal. The thickness and echogenicity of kidney parenchyma are important in predicting kidney function, and dysplastic parenchyma in severe cases is often a sign of a poor prognosis [12].

Oligohydramnios is one of the most important prognostic parameters in fetal life. It may be quantified by measuring the AFI (amniotic fluid index). An AFI < 5 cm is defined as cut-off for the diagnosis of oligohydramnios [15]. In combination with bilateral hydronephrosis and dysplastic kidney parenchyma, it also reflects an indicator of a poor prognosis. Prenatal intervention to drain the hydronephrosis may be an option, but there is not much literature on this topic. Shimada et al. mention fetal puncture in three patients in a small series of children with giant hydronephrosis, but do not discuss patients’ outcomes [8].

Therefore, prenatal counseling in these patients is difficult. All the factors mentioned above will not predict the final postnatal outcome [16,17]. Despite the huge hydronephrosis, the absent kidney parenchyma on ultrasound and oligohydramnios before birth, our patient had a very good postnatal outcome with normal kidney function.

The gold standard surgical treatment of UPJO is still the Anderson–Hynes pyeloplasty. There is some literature—describing nephroplication of huge dilated renal pelvis during pyeloplasty procedure in patients with giant hydronephrosis—with good success rates being reported [18,19,20]. A modified variant of nephroplication is a y-plasty, as described by Belman et al. [21]. Another study of Ansari et al. proposes ureterocalicostomy in UPJO with giant hydronephrosis as a valuable alternative to pyeloplasty with excellent outcomes [22]. We resected a part of the kidney pelvis and performed a traditional Anderson–Hynes pyeloplasty without nephroplication or y-plasty. Our patient needed percutaneous nephrostomy tubes for several months on both sides, which then could be removed without further measures. We think that the highly enlarged kidney pelvis needed this time to recover and recover some elasticity. It remains unclear if the duration of this tube drainage could have been shortened by performing nephroplication.

Despite UPJO neither necessarily being associated with additional malformations nor part of a syndrome, hydronephrosis may be an associated feature of multiple malformation syndromes including VACTERL, Schinzel–Gidion syndrome, Johanson–Blizzard syndrome, Ochoa syndrome or trisomy 13/18 [23]. There are also descriptions of giant hydronephrosis in patients with Soto’s syndrome [24]. Our patient showed minor syndromic features (head deformity and high myopia), but the genetic investigations remained unremarkable until today.

## 4. Conclusions

Bilateral giant hydronephrosis in a newborn is a very rare condition compared with relatively common unilateral dilation. Although this anomaly is likely to be associated with a poor prognosis, our case report shows a good outcome, including well-preserved kidney function. Even in kidneys with a bad split function, there is a potential for some recovery. Prenatal counseling should be perfomed carefully with discussion of different possibilities and without definitive prediction of outcome.

## Data Availability

Not applicable.
